# Defining Life Skills in health promotion at school: a scoping review

**DOI:** 10.3389/fpubh.2023.1296609

**Published:** 2023-12-07

**Authors:** Adeline Darlington-Bernard, Corélie Salque, Julien Masson, Emily Darlington, Graça S. Carvalho, Florence Carrouel

**Affiliations:** ^1^Laboratory Health Systemic Process (P2S) UR4129, University Claude Bernard Lyon 1, University of Lyon, Lyon, France; ^2^Laboratory Education, Cultures, Politics, University Lumière Lyon 2, Lyon, France; ^3^CIEC, University of Minho, Braga, Portugal

**Keywords:** health promotion, schools, Life Skills, definition, terms, concept, French, English

## Abstract

**Background:**

Life Skills have been central to Health Promotion interventions and programmes with children and adolescents for over 40 years. School is a strategic setting for Life Skills education. Recently, policy-and decision-makers have focused on Life Skills development for youth. Research on Life Skills has gained momentum. Different terms are used to discuss and define Life Skills. Research identifies a lack of conceptual definition. The purpose of this study is to identify the definitions in the literature in English and French, and to reach a conceptual and consensual definition.

**Method:**

The Scoping Review methodology was used. Three research questions aim to identify how Life Skills are defined in the field of health promotion at school, to see whether a conceptual and consensual definition exists, and, if relevant, to propose a conceptual definition. The search was conducted in 5 databases by 3 reviewers. This study focused on full-text publications in English or French, human studies, health promotion in school, school pupils, teacher training, and with a definition of Life Skills. Publications on after-school activities, higher education outside teacher training, adult education, other than peer-reviewed scientific papers were excluded.

**Results:**

48 publications were included in English and 7 in French. NVIVO was used to determine and compare the French and English terms used for Life Skills and their definitions. According to the three research questions, (i) the terms used to define Life Skills are diverse and numerous, with different purposes at school in relation to health promotion, and different taxonomies, and relate to different areas of research; (ii) no consensual, conceptual definition of Life Skills was found; (iii) further semantic, epistemological and ontological clarifications are required.

**Conclusion:**

Some conceptual definitions of Life Skills exist without consensus. Life Skills being at the crossroads between different fields could explain this and is illustrated by the multiplicity and diversity of the terms employed, and the various taxonomies and purposes used at school in health promotion. This may also explain why they are difficult to evaluate. Defining Life Skills consensually cannot be achieved due to the diversity of research perspectives from different fields.

## 1 Introduction

In association with information provision and education, Life Skills have been identified by the World Health Organisation (WHO) as an enabling factor in supporting health promotion action, through the development of personal skills, notably through educational settings. In 1993, WHO proposed their own official definition of Life Skills as being “the abilities for adaptive and positive behaviours that enable individuals to effectively deal with demands and challenges of everyday life” (1, 2). This initial definition comprised a set of 10 life skills divided into 5 pairs (1): (i) decision-making and problem-solving, (ii) creative thinking and critical thinking, (iii) self-awareness, and empathy, (iv) communication skills and interpersonal relationship skills and (v) coping with emotions and managing stress.

Since then, Life Skills have been explored and added to. A taxonomy was notably proposed by Mangrulkar et al. in 2001 (3): they categorised Life Skills into 3 groups, namely social, cognitive and emotional skills. This taxonomy has been particularly used in France, by the Instances Régionales d’Education et de Promotion de la Santé (IREPS - regional bodies for health education and promotion). In parallel, other non-governmental Organisations (NGO) have also proposed their own definition and taxonomies of Life Skills: while the United Nations Educational, Scientific and Cultural Organisation (UNESCO) propose a wider definition of Life Skills as being *a mix of knowledge, behaviour, attitudes and values and designate the possession of some skill and know-how to do something or reach an aim. They include competencies such as critical thinking, creativity, ability to organise, social and communication skills, adaptability, problem solving, ability to co-operate on a democratic basis that are needed for actively shaping a peaceful future* (4), the United Nations International Children’s Emergency Fund (UNICEF) either refer to the definitions by WHO, or to that which they proposed in 2000 as *psycho-social and interpersonal skills used in every day interactions…not specific to getting a job or earning an income* (5). Both definitions proposed by UNESCO and UNICEF remain more general and make no reference to health: the first focuses on peace as an end, whereas the second centres on *interactions* and the social dimension of life skills, and insists on their general nature. In 2021, WHO issued an updated version of its health promotion Glossary of terms (6), in which Life Skills appear more strongly connected to health than ever. Indeed, the Life Skills entry shifted from *Life Skills* to *Skills for Health (Life Skills)* (6). Moreover, in the literature, Life Skills are identified as being a health-protecting factor (7), notably in primary prevention with health-compromising behaviour (8, 9) mental health (10) and violent and risky sexual behaviour (3). In addition, Life Skills development appears to foster well-being, sociability, positive social interactions, cognitive development, academic achievement, and professional success (11–13). It is also recommended they be developed through participation and experiential pedagogical methods (11, 14), through integrated and intersectional approaches (11, 12). Thus, Life Skills seem to be a dynamic notion, which has been evolving over time, with many identified benefits for health and well-being, through multiple approaches.

School is a key setting for health promotion, as it provides access to a wide population of children. It is also a means to educate pupils from an early age in developing health knowledge and healthy behaviours, as well as to tackle social determinants of health inequality (2, 15–17). Healthy school environments play an important role in physical, social, emotional and cognitive development. For instance, school-based physical activity can improve overall health in all pupils and encourage the development of healthy lifestyles (18, 19), especially with children who would not otherwise have access of sports outside of school. School can also be a place to promote healthy eating habits through a whole-school approach thanks to healthy school meals and nutrition education (20). Furthermore, school is the place where pupils acquire knowledge and learn how to use it (i.e cognitive development), live alongside others and learn how to behave in a group and in society (i.e social development) and where they are placed in situations through which they will have to learn about themselves and work on dealing with their emotions (i.e emotional development) (21). In addition, developing Like Skills through school provides a support system for adolescents (22) with documented benefits in terms of education, prosocial behaviour, health, culture, the economy, employment as well as for society and the individual (23). Thus Life Skills education and development in school settings have been the focus of growing interest by policy-and decision-makers, for example in France, with the progressive and explicit integration of health promotion principles and Life Skills development in the Education Nationale School Curriculum and guidelines (24). For all this, a lack of clarity has been highlighted in the literature. Indeed, a global perspective is needed on the terms and definitions used (7). Worse, Life Skills may be considered as a *fourre-tout*, i.e., a *hotchpotch* concept (25). Further epistemological clarification seems to be required, first and foremost regarding the definition of Life Skills in the field of health promotion at school. Thus, the objective of this study is to explore the literature to determine both in French and English (i) how Life Skills are defined in the field of health promotion at school, (ii) to find a conceptual and consensual definition, and (iii) if relevant, to propose a conceptual definition.

## 2 Materials and methods

A literature review based on the scoping review principle was performed to provide an overview of the scientific data (26, 27). The scoping review format was preferred over the systematic review format as the information on the topic studied was complex and diverse (28). The manuscript was reported following the PRISMA-ScR criteria (Preferred Reporting Items for Systematic reviews and Meta-analyses) (Supplementary Table S1) (29). The scoping review was conducted guided by the five-step methodological framework from Arksey et O’Malley (26): (i) identification of a specific research objective and search procedures, (ii) identification of pertinent publications, (iii) selection of publications, (iv) data extraction, and (v) summary, analyses and reporting of results.

### 2.1 Research questions

In the field of health promotion at school, the research questions were:

(i) What are the terms and definitions of Life Skills used in French and in English?
(ii) Does a conceptual and consensual definition exist?
(iii) If not, can a conceptual definition of Life Skills be proposed?

### 2.2 Identification of pertinent publications

#### 2.2.1 Database search

The search was conducted from December 2022 to January 2023 using 5 databases: Google Scholar, ERIC, Pubmed, ISIDORE and HAL SHS. The following search terms were used: in English (“life skills” OR “Socio-Emotional Skills” OR “basic skills” OR “problem-solving learning” OR “skills for health”) AND (“literature review” OR “systematic review” OR “definition” OR “education”); in French, (“compétences psychosociales” OR “compétences individuelles indispensables à la vie” OR “habiletés ou aptitudes psychosociales” OR “développement psychosocial”) AND (“revue de littérature” OR “revue systématique” OR “définition” OR “éducation”). Two reviewers performed this research and removed the duplicates.

#### 2.2.2 Screening and eligibility of publications

The inclusion criteria were: (i) publications written in the English or French language; (ii) publications presenting human studies; (iii) publications focusing on health promotion at school; (iv) publications focusing on school pupils; (v) publications focusing on teacher training; (vi) publications which include a definition of Life Skills. The exclusion criteria were: (i) publications focusing on after-school activities; (ii) publications focusing on higher education outside teacher training; (iii) publications focusing on adult education; (iv) publications other than peer-reviewed scientific papers such as conference papers, book extracts, institutional reports, congress abstracts or commentaries.

The screening of studies was carried out independently by the two reviewers based on an examination of the titles and abstracts identified in the electronic databases. Selected publications were cross-checked, and any discrepancies were resolved through discussion to reach consensus on study inclusion.

### 2.3 Selection of publications

The full text of the selected publications was analysed by the two reviewers who retained only the publications that answered the research question. As before, in case of divergence, the reviewers discussed until they reached a consensus.

### 2.4 Collection, summary and reporting of results

A narrative synthesis was made from the publications included. A narrative synthesis is a process which may be used in exploratory studies on a large array of questions. It does not solely focus on the effectiveness of interventions (5). This approach involves synthesising the results of several studies and is based primarily on the use of words and text to summarise and explain the results of the synthesis. An inductive approach of the data was chosen, to allow the text corpus to “tell the storey” of how Life Skills are defined in the literature. Then, the data collected was analysed using the NVIVO qualitative data analysis software (QSR International Pty Ltd. Version 12, 2018). Each definition was encoded in NVIVO, to identify and categorise the terms and references used to define Life Skills. This review involved 3 researchers (ADB, CS, FC) to ensure strong review validity. They were involved in discussions and decisions pertaining to the selection of search terms, inclusion and exclusion criteria and publication selection and analysis. The other three researchers (ED, GC & JM) proofread the article before submission and provided support throughout the review process.

### 2.5 Data extraction from the included studies

For each publication included, key information was reported in a series of figures and tables. They included the authors, the year of publication, the definition of Life Skills, and the references of the definitions found.

## 3 Results

### 3.1 Overview of publications included

**Figure 1:**
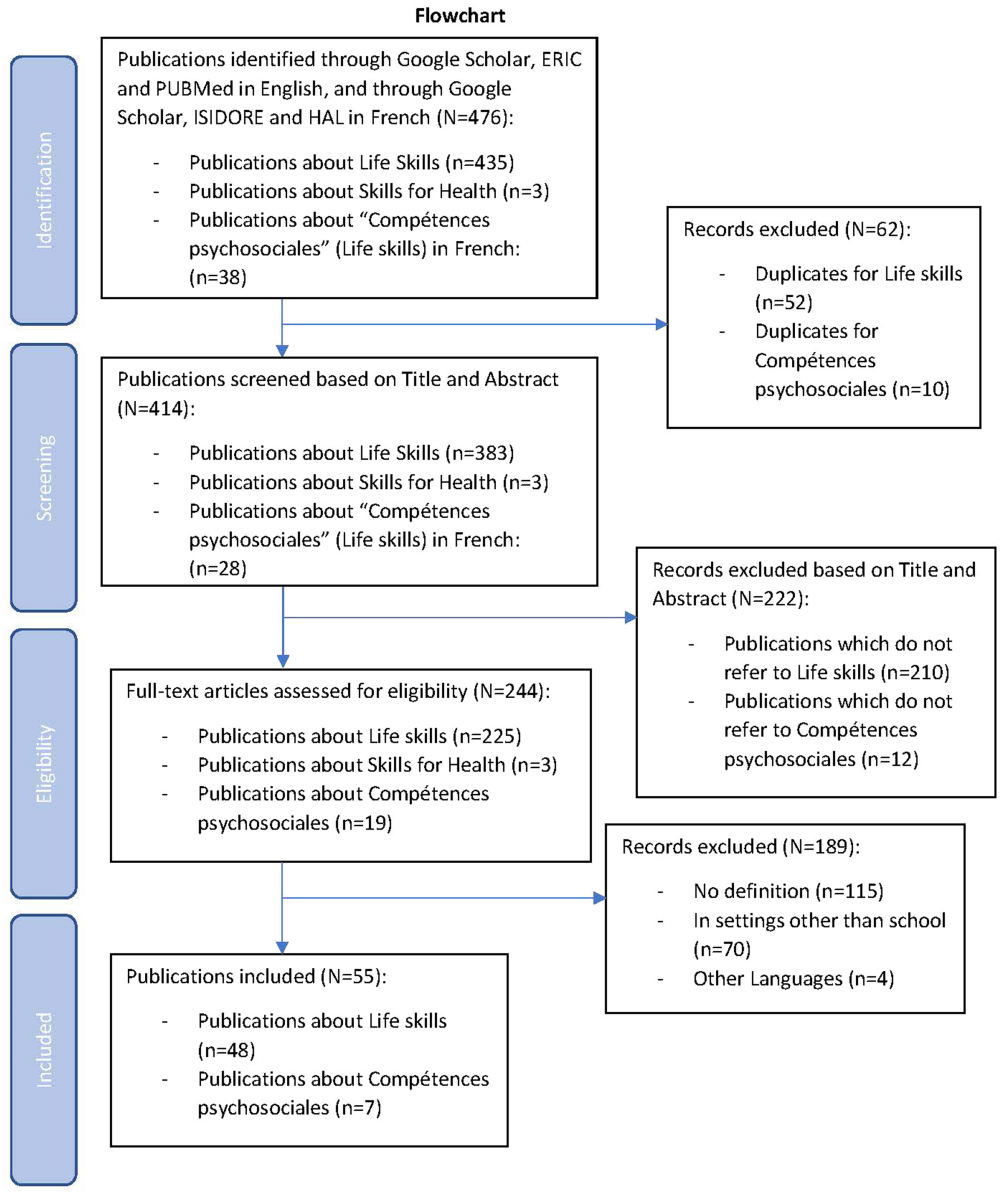
Flowchart of scoping review process (PRISMA-ScR).

A total of 476 peer-reviewed publications were identified: 438 in English and 38 in French. 414 of these were screened based on title and abstract after removal of the 62 duplicates: 383 publications about Life Skills in English, 3 about Skills for Health, and 28 publications about *Compétences Psychosociales* in French remained. Eligibility was established for 244 publications, with 228 publications in English and 19 in French. Finally, 55 publications were included as matching the criteria for our scoping review, i.e., 48 publications in English about Life Skills and 7 publications in French about *Compétences psychosociales*. 115 publications were excluded as they included no definition of Life Skills, Skills for Health or Compétences Psychosociales, 70 publications were excluded as they explored other settings than school and 4 publications proposed an abstract in English with the subsequent text in another language than English or French (Figure 1).

### 3.2 Papers included

Overall, 55 papers were analysed: 48 in English and 7 in French. The 55 publications selected and included in the review dealt with Life Skills education, interventions, programmes, or development at school. The papers identified were published between 2008 and 2022 and were coded from P1 to P55 (Table 1).

**Table 1:** Repartition of included papers per year and per language.

Year of publication	Papers included in English		Papers included in French
2022	P1 (30) P2 (31) P3 (32) P4 (33) P5 (34)	P6 (35) P7 (36) P8 (37) P9 (38)	
2021	P10 (39) P11 (40) P12 (41) P13 (42) P14 (43)	P15 (44) P16 (45) P17 (46) P18 (47)	P49 (48) P50 (7) P51 (49) P52 (50)
2020	P19 (51) P20 (52) P21 (53)	P22 (54) P23 (55)	P53 (56)
2019	P24 (57) P25 (58) P26 (59)		
2018	P27 (60) P28 (61) P29 (62)	P30 (63) P31 (64)	
2017	P32 (65) P33 (22)		P54 (66) P55 (67)
2016	P34 (68) P35 (69) P36 (70)	P37 (71) P38 (72)	
2015	P39 (73)		
2014	P40 (74) P41 (75) P42 (76)		
2013	P43 (77) P44 (78)		
2010	P45 (79) P46 (80)		
2008	P47 (81) P48 (82)		

Henceforth, the term “paper” will be used to refer to the articles included in the results of the scoping review; the term “article” will be used to refer to the articles cited as reference to provide a conceptual definition of Life Skills; finally, the term “publication” will be used to refer to all other references.

#### 3.2.1 Definitions of Life Skills by NGOs

In English, 31 papers referred to the definitions established by NGOs (Table 2). Five WHO publications (10, 15, 83, 85, 86) were cited in 24 papers included (P1, P3, P6, P8, P10, P11, P14, P16, P17, P18, P19, P21, P22, P28, P29, P33, P34, P39, P40, P44, P45, P46, P47, P48). One UNICEF publication (84) was cited in 4 papers included (P3, P14, P17, P29). Other NGO sources comprised Organisation for Economic Co-operation and Development (OECD) and the United Nations (UN).

**Table 2:** NGO sources cited as references to define Life Skills in the papers included in English.

NGO	Title of NGO publications cited in papers included in English	Year of publication	Papers included in English
*WHO*	Life Skills education in schools (83)	1997	P3, P14, P16, P28, P29, P33, P34, P39, P45, P46, P47
*WHO*	Skills for health: Skills-based health education including life skills: an important component of a child-friendly/health-promoting school (10)	2003	P6, P11, P18, P21, P22
*WHO*	Life Skills education in schools (13)	1993	P6, P10, P21, P40, P44
*UNICEF*	Global Evaluation of Life Skills Education Programmes (84)	2012	P3, P14, P17, P29
*WHO*	Partners in Life Skills Education. Conclusions from a United Nations Inter – Agency Meeting (15)	1999	P17, P19, P48
*WHO*	Life Skills education school handbook: Prevention of noncommunicable diseases: approaches for schools (85)	2020	P1, P8, P18

As shown in Table 2, WHO publications are the most cited NGO source to define Life Skills in English among the papers included in this study. It is also the case regarding publications in French: all 7 papers included references to definitions by WHO. Three papers (P49, P50, P51) cited “Life Education in Schools” (1994) (13) and 2 papers (P51, P53) cited “Skills for health: skills-based health education including life skills: an important component of a child-friendly/health-promoting school” (2003) (10).

#### 3.2.2 Conceptual definitions of Life Skills in the papers included in the scoping review

In English, 23 papers included in English cited articles which proposed conceptual definitions of Life Skills (Table 3). The 9 articles cited more than once, were published from 1995 to 2014. Furthermore, the most cited article (5 times) was Danish et al. (87).

**Table 3:** Articles cited more than once, which include a conceptual definition of Life Skills in English in papers included in English.

Papers included in English	Articles cited which include a conceptual definition of life skills	Field
P18, P20, P32, P36, P35	Danish et al. (2004) (87)	Sports psychology
P18, P33, P47	Danish & Nellen (1997) (88)	Sports psychology
P6, P20, P47	Hodge & Danish (1999) (89)	Psychology
P30, P33	Hodge et al. (2013) (90)	Sports psychology
P18, P33	Danish & Donohue (1995) (91)	Sports psychology
P30, P32	Kennedy et al. (2014) (92)	Health psychology
P19, P20	Kolburan & Tosun (2011) (93)(unavailable in English)	Psychology
P10, P31	Srikala & Kishore (2010) (80)	Psychology
P1, P39	Smith et al. (2004) (94)	Psychology

As regards the conceptual definitions found in French, 2 papers included in the scoping review referred to scientific sources spanning from 2001 to 2021. In P54 by Encinar et al. (66), the 4 international references cited spanned from 2001 to 2010 and included Mangrulkar et al. (3), Danish et al. (87), Gould et al. (95), and Goudas (96). In P52 by Simar et al. (7), the 6 scientific articles cited include Fonte et al. (97), Fanchini (98), Saugeron et al. (25), Gorza et al. (99), Morlaix and Fanchini (100), and Morlaix et al. (48).

**Figure 2:**
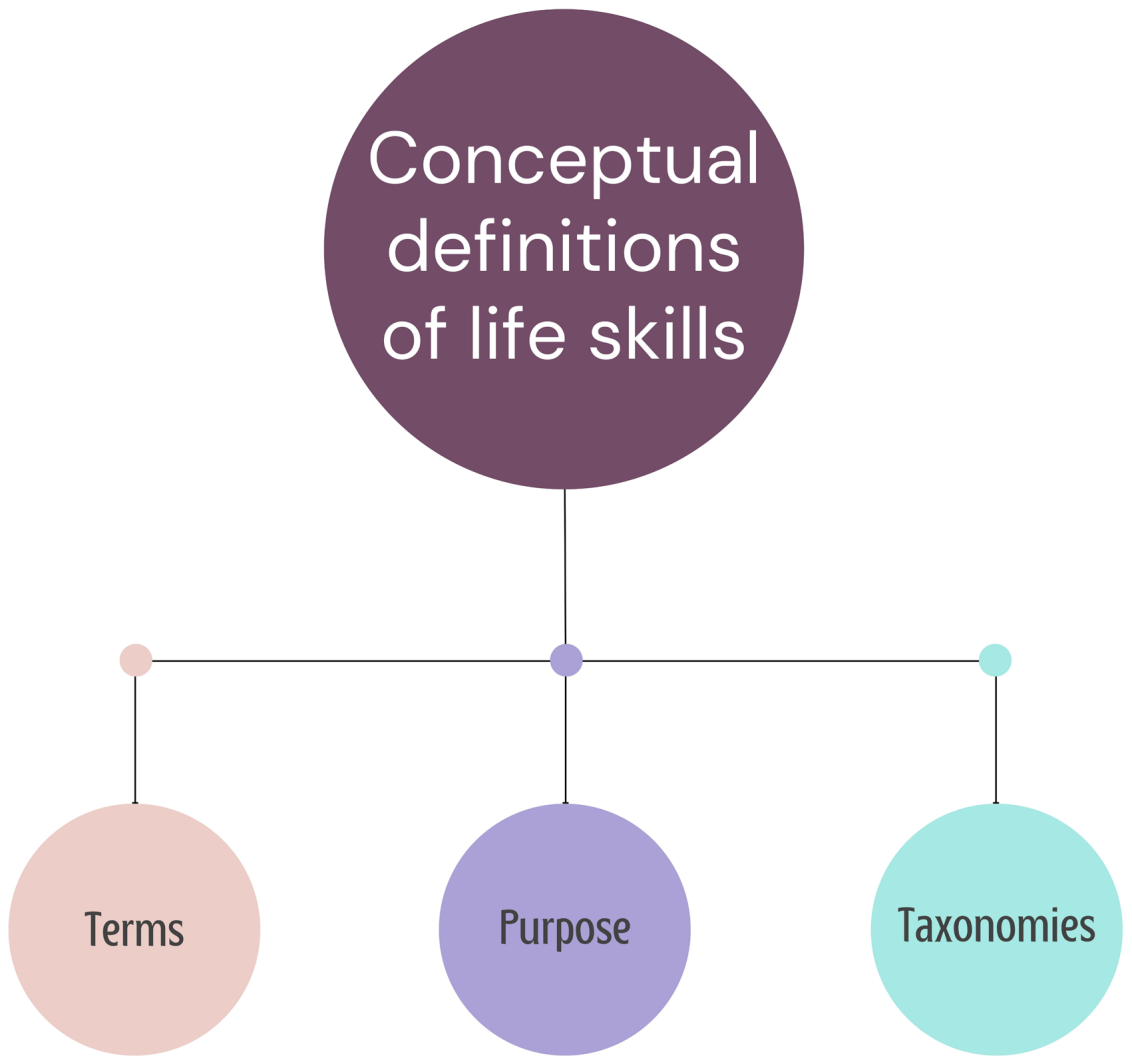
Categories established for the analysis of the conceptual definitions of Life Skills through QSR International’s NVIVO.

Analysis of the conceptual definitions of Life Skills through QSR International’s NVIVO allowed the identification of the three categories (Figure 2): (i) terms, (ii) purpose, and (iii) taxonomies. While this Scoping review does focus on Life Skills in the field of health promotion at school, the 3 categories established remain rather general. They will be described below.

##### 3.2.2.1 Terms used to describe Life Skills in the conceptual definitions

**Figure 3:**
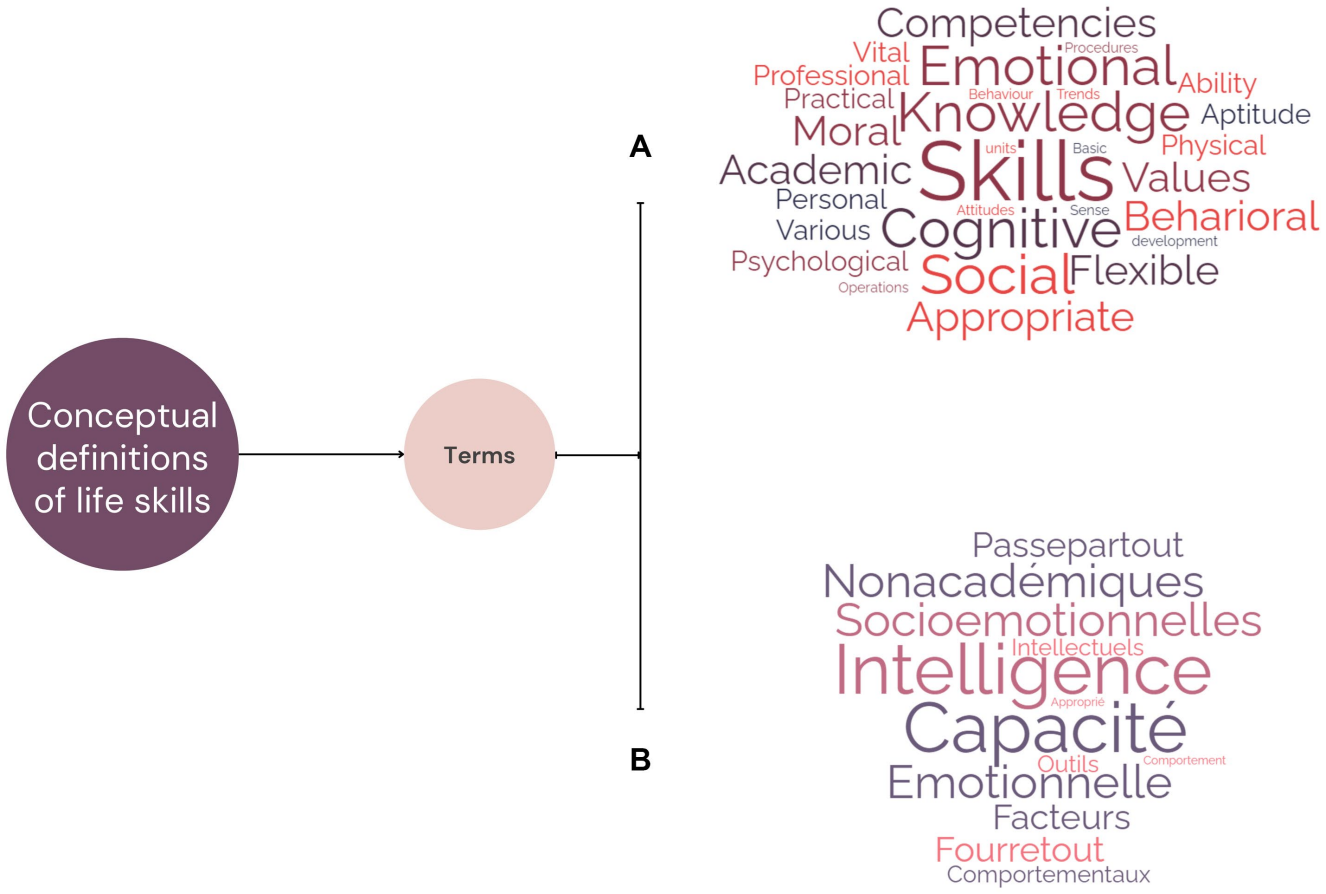
Terms (nouns and adjectives) used to describe Life Skills in the conceptual definitions found in the papers included in English **(A)** and in French **(B)**.

The nouns and adjectives used, in English and in French, to describe Life Skills in the conceptual definitions found in the papers included for analysis are presented in Figure 3. They are shown in the form of two word clouds: one in English and the other in French.

The 5 nouns most used in English were *skills* (in 11 included papers), *knowledge* (in 3 included papers), *competencies* (in 2 included papers), *values* (in 2 included papers) and *pool* (in 2 included papers). In terms of adjectives, the most cited were *emotional*, *cognitive* and *social*. The 2 nouns most used in French were *compétences* (skills, competencies, in 3 included papers) and *capacité* (capacity, ability, in 2 included papers). The adjective *comportemental* (behavioural) was also cited in 3 included papers.

A list of associated terms was also compiled from the papers included in English (Table 4).

**Table 4:** Terms associated with or used as synonyms of life skills in the papers included in English.

Associated Terms	Papers included in English
21st century skills	P15, P23, P28
Behavioural psychological learning ability	P14
Characters skills	P15, P23
Cognitive skills	P11
Communication skills	P16
Interpersonal competencies	P3
Interpersonal skills	P16, P32
Non-cognitive skills	P15, P23
Personality traits	P15, P23
Positive attitudes and skills	P3
Psychological and interpersonal skills	P29
Psychological and social skills	P3
Psychological skills	P14
Psychosocial abilities	P27
Psychosocial and behavioural abilities	P18
Psycho-social competence	P3, P36
Psychosocial competencies	P6, P16
SEL / Social and Emotional Learning	P4, P15, P23, P34
Socio-Emotional skills	P8, P11, P15
Soft skills	P15, P23
Temperament	P15, P23
Transferable skills	P8

A similar list could not be compiled from the papers included in French, as no other associated terms were found.

##### 3.2.2.2 Purpose of Life Skills in the conceptual definitions

**Figure 4:**
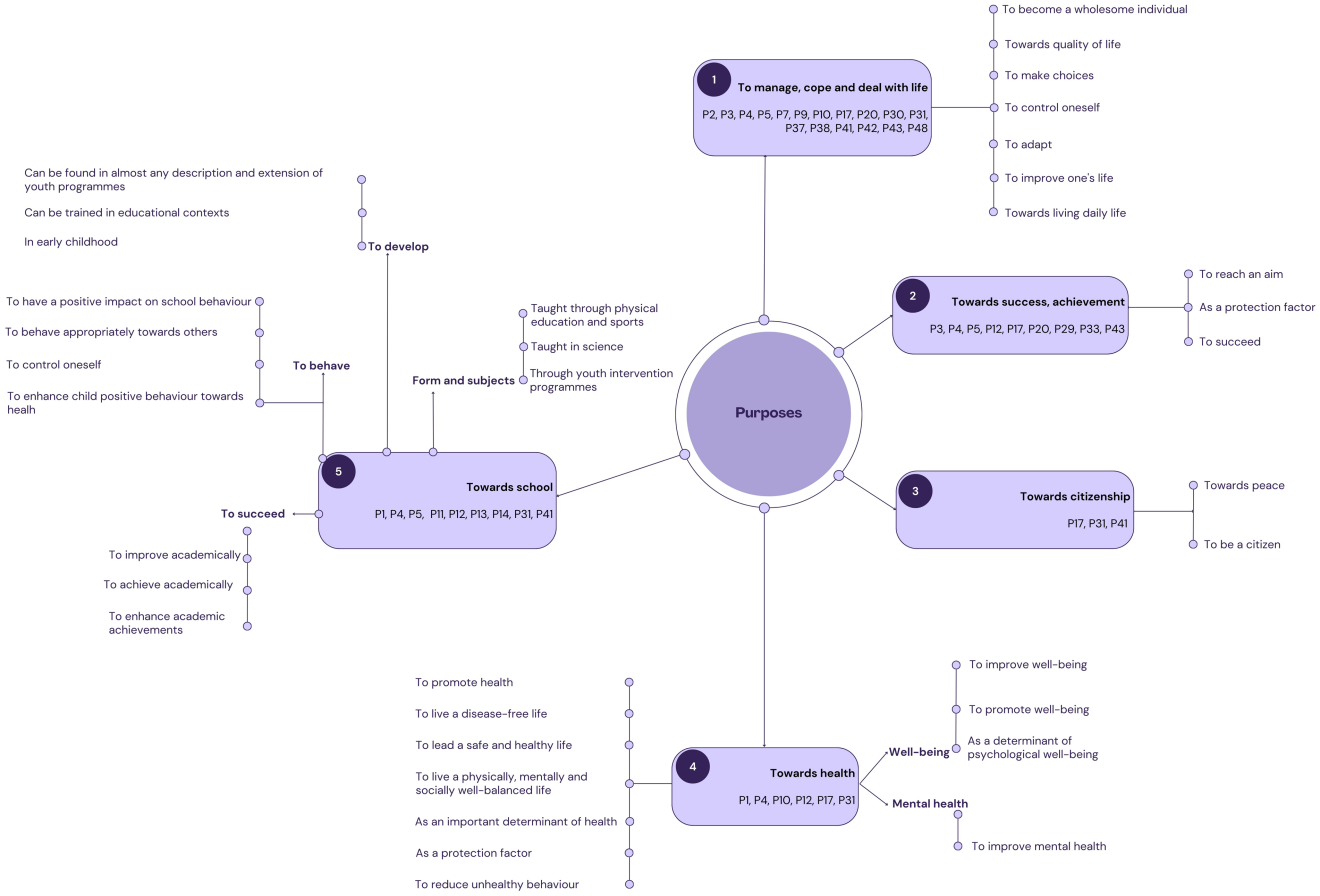
Purpose of Life Skills based on the conceptual definitions found in the papers included for analysis in English.

One of the aspects mentioned in the conceptual definitions found in the papers included for analysis was the purpose for which Life Skills were being developed in a school setting in a health promotion perspective. These purposes were categorised under 5 topics (Figure 4): (1) *To manage, cope and deal with life*, (2) *Towards success, achievement*, (3) *Towards citizenship*, (4) *Towards health* and (5) *Towards school*. The topics entitled *Towards health* was further sub-categorised into three areas: *Well-being*, *Mental health* and a more general *Health* category. *Towards school* was created to explore Life Skills in the service of school more specifically. Three sub-categories were established to record the purpose of Life Skills in the service of school: (1) *To succeed*, (2) *To behave*, (3) *To develop*. The 4th sub-category was created with elements regarding form or the school subject through which Life Skills were being developed: (4) *Form / School subject*.

In French, the purposes of life skills identified in the conceptual definitions used in the papers included for analysis were categorised into three topics: towards managing, dealing and coping with life, especially in terms of adaptation; towards interactions, with one’s environment, with others, and exerting a positive influence on oneself and others; towards health, to promote well-being and prevent health-compromising behaviours.

##### 3.2.2.3 Taxonomies of Life Skills from the conceptual definitions

**Figure 5:**
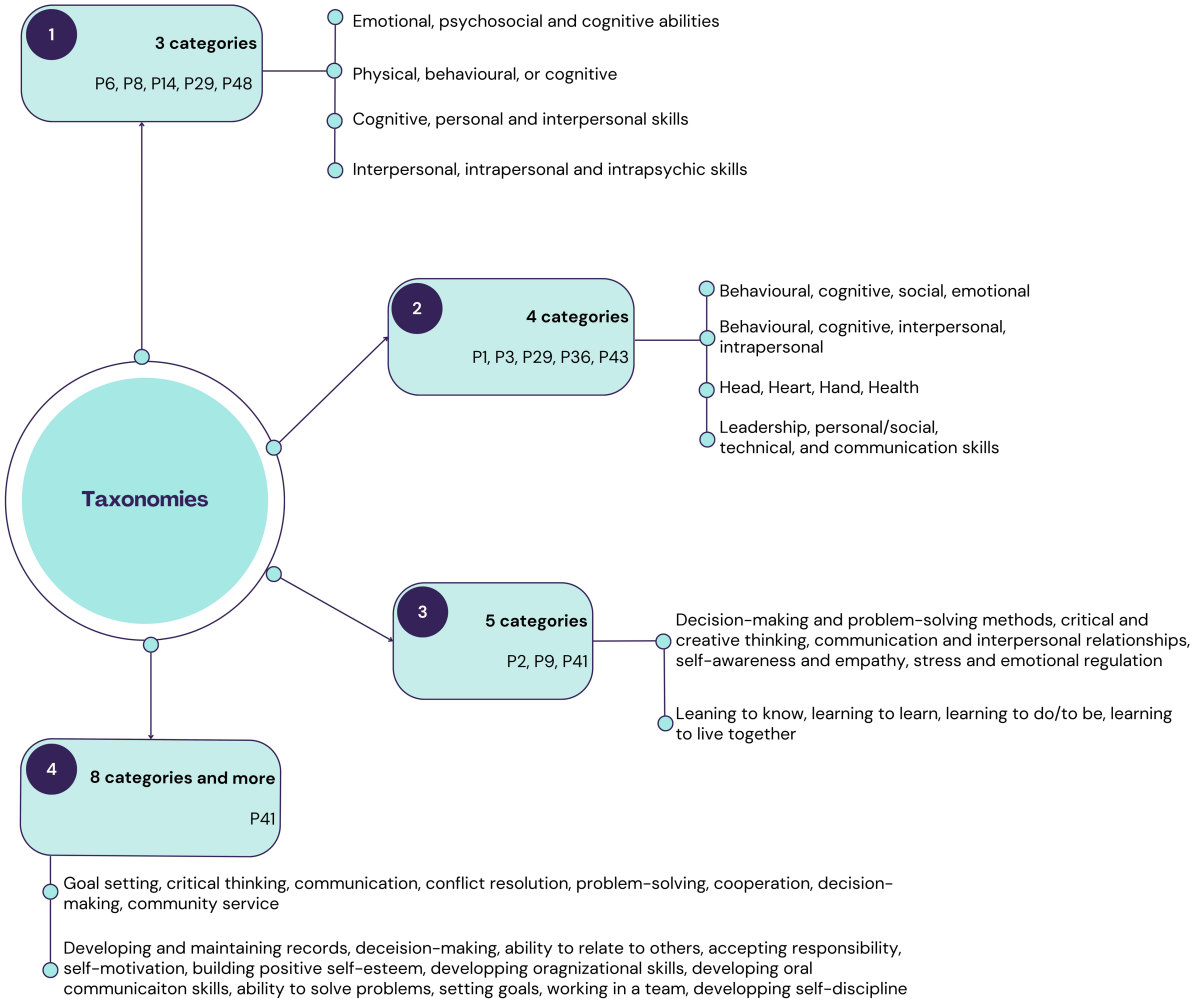
Life Skills taxonomies identified in the conceptual definitions in English.

The different taxonomies listed from the conceptual definitions identified in English are presented in Figure 5. The taxonomies were categorised under 4 topics: (1) taxonomies with 3 categories, (2) taxonomies with 4 categories, (3) taxonomies with 5 categories, (4) taxonomies with 8 categories and more.

Topics 1 and 2 refer to general types of Life Skills in the field of Health Promotion at school. Topics 3 and 4 gather taxonomies which propose detailed and complex declensions of life skills: they are more operational and practical to be developed and applied in health promotion at school.

In the papers included in French, no specific taxonomies were detailed in the conceptual definitions found.

## 4 Discussion

This Scoping Review was conducted in both English and French to collect and analyse the conceptual definitions of Life Skills in the field of health promotion at school. Currently, Life Skills education and development is gaining momentum in international research, and more specifically in France (101, 102). Indeed, Life Skills development at school, especially in a health-promoting perspective, is being reinforced through policy. It is also a growing focus for research. This article was thus the opportunity to see which terms and definitions are used in international and francophone research.

First, the findings show some definitions of Life Skills exist. Some are proposed by NGOs, others provided by the scientific community. These definitions are often reworded and sometimes aggregated to form a mix between the operational and conceptual (33, 72, 73, 82). Although the conceptual definitions of Life Skills are numerous (63, 90), the difficulty to establish a consensual, conceptual definition has been identified (63, 66). Our findings tend to confirm that no specific conceptual definition is consensual. Indeed, the definitions provided by WHO over the years still remain significantly cited when Life Skills are defined in the literature as being “Life Skills are abilities for adaptive and positive behaviour, that enable individuals to deal effectively with the demands and challenges of everyday life” (1, 83). One explanation for the lack of a consensual, conceptual definition of Life Skills may lie in the fact that Life Skills have been the focus of recent emphasis (36). However novel the study of Life Skills may be, this argument might not suffice to justify a consensual definition could not be found. Another explanation for the lack of consensus may lie in the breadth in scope of the terms used to define Life Skills. According to our findings, the terms highlighted in both English and French show the connections of Like Skills with the fields of Education (e.g., *academic*, *competencies*, *aptitude*, *skills* in English; *compétences* (skills, competencies), *capacité* (capacity, ability), *outils* (tools), in French) and Psychology (e.g., *psychological*, *behavioural*, *cognitive*, *behaviour* in English; *comportement* (behaviour), *comportementaux* (behavioural), *intellectuels* (intellectual) in French). The conceptual definitions of Life Skills also seem to lean towards ties with the field of Sociology. Yet, it is through health promotion that Life Skills become a strategic factor for primary prevention (3, 9, 103) and thus gain international focus, notably through WHO (1, 2, 6). This fluctuation between different fields is further reinforced through the declension of Life Skills found in the different taxonomies. Moreover, it seems that when Life Skills are being developed in school, health is not the sole objective. While 4 general purposes have been identified in connection with health promotion in school settings (i.e., to manage, cope and deal with life, towards success and achievement, towards citizenship and towards health), our findings also tend to show that Life Skills development at school focuses on education, and more specifically on learning and succeeding. Thus, Life Skills appear connected to a complex entanglement of dimensions which includes *cultural* (63, 68, 76), *environmental* (68) or *psychological* and *demographic factors* (32). Avci et al. (32) cite *gender*, *grade level*, *grade point average (GPA) or academic performance*, *socio-economic status* and *well-being* as examples. Simar et al. also refer to *human capital indicators*, including *cultural capital*, *social capit*al and *emotional capital* (7) which may be used as a reference to establish a definition of Life Skills.

From a linguistic and semantic point of view, another observation can be drawn from the findings of this Scoping Review: it shows the multiplicity and diversity in the terms used to define Life Skills conceptually in the field of health promotion at school. This can also be noted through the purpose for which Life Skills are being developed in a school setting, and more specifically towards school, as well as in the taxonomies of reference used for Life Skills, which range from 3 to 8 Life Skill categories. Our findings provide an overview of the associated terms used to refer to Life Skills. It is worth noting that synonyms of the word *skills* are used to refer to Life Skills, such as *ability*, *aptitudes*, *competencies*, *competences*, *attitudes*, or *traits*. But these terms are not synonymous. For instance, an *aptitude* is “the capacity to acquire competence or skill through training” (104). It is thus different from an *ability*, which is defined as an “existing competence or skill,” which “may be either innate or developed through experience” (104). Additionally, an *attitude* is “one’s developed repertoire of skills, especially as it is applied to a task or set of tasks. A distinction is sometimes made between competence and performance, which is the extent to which competence is realised in one’s actual work on a problem or set of problems” (104). Whereas a *trait* is “an enduring personality characteristic that describes or determines an individual’s behaviour across a range of situations” (104). As for adjectives, they mostly revolve around personality, learning or social interactions. A similar overview of synonyms was proposed by Santé Publique France, in their 2021 scientific report on Life Skills (102). It shows *compétence* (skill, competency) to be the preferred term, to which a wide array of adjectives is added as descriptors, the dimensions of which are either emotional, social and cognitive dimensions. Life Skills are thus perceived as being a *polysemic and polymorphic notion* (7, 97), as well as an *all-purpose* and a *hotchpotch* term (7). The use of synonyms to refer to Life Skills thus seems rather common, notably in French: as Encinar et al. highlights, they are used interchangeably (66). Another linguistic point of interest may also be discussed regarding the translation of *Life Skills* into French, i.e., *compétences psychosociales*. It can be noted that the term *skills* becomes *compétences* in French, while the qualifying notion of *life*, which expresses the purpose of Life Skills, seems totally lost in translation, as it becomes *psychosociales*, focused on the technical nature of *Compétences Psychosociales*. This observation also reinforces a lack of consensus in the terms used to discuss Life Skills and the difficulty to propose a consensual translation and definition. However, this particular point does not seem to have been discussed in the literature, probably due to the fact that French is not the language of reference for international research.

This scoping review and its results might thus contribute to explaining why Life Skills are so difficult to define and why a conceptual framework has not yet been established (68, 90). This may also have an effect on the evaluation and measurement of Life Skills development, notably with schools pupils: so far, Sancassiani (73) explains no consensual method or tools has been developed to measure Life Skills.

Finally, this scoping review has several strengths. Firstly, it was conducted in accordance with the PRISMA-Sc methodology. Secondly, it seems it is the first review to propose an analysis of the definitions of Life Skills. Thirdly, this scoping review was conducted in two languages and focuses on English and French terms. However, the following weaknesses have also been identified. First, the research was conducted in 5 databases, but others could have been used such as Scopus. The databases were chosen since they were made available by our institution. Additionally, some of the references we found could not be accessed freely. Some articles were thus not included due to this reason. Secondly, the imbalance between the number of papers included in French and in English is noticeable. Indeed, while international research on Life Skills has been developing over more than 30 years, it is not the case in France. Finally, due to the language barrier, many papers were not included in this study. It would have been interesting to have a broader range of papers, as Life Skills might be defined differently from other cultural perspectives.

## 5 Conclusion

According to the three research questions of this study, (i) the terms used to define Life Skills are diverse and numerous, with different purposes at school in relation to health promotion, and different taxonomies, and they also appear to relate to different areas of research; indeed, they are mostly connected to the fields of psychology, education, and health promotion, and they are geared towards dealing with life and its challenges, as well as attaining more specific purposes; (ii) some conceptual definitions of Life Skills can be found in the literature, in both French and English; however, no consensus was found in terms of a conceptual definition of Life Skills; (iii) consequently, defining Life Skills consensually from a conceptual point of view cannot be achieved due to the diversity of the areas of research, which have different perspectives, and adopt different purposes and approaches.

Further epistemological, ontological and linguistic clarifications seem to be required in order to clearly establish the scientific grounds of Life Skills, notably in terms of field of origin and rooting, and in terms of scope, especially around the notion of skill: while Life Skills development and education are recognised as a valuable tool in the service of health, education and social interaction, perhaps it is worth questioning the fact that *Life Skills* is in fact a broad concept.

## Author contributions

AD-B: Conceptualization, Formal analysis, Methodology, Project administration, Validation, Visualization, Writing – original draft, Writing – review & editing. CS: Formal analysis, Writing – review & editing. JM: Writing – review & editing. ED: Writing – review & editing. GC: Writing – review & editing. FC: Conceptualization, Methodology, Project administration, Validation, Writing – original draft, Writing – review & editing.
